# Palladium nanoparticles decorated into a biguanidine modified-KIT-5 mesoporous structure: a recoverable nanocatalyst for ultrasound-assisted Suzuki–Miyaura cross-coupling

**DOI:** 10.1039/c9ra08809a

**Published:** 2019-12-16

**Authors:** Hojat Veisi, Amin Mirzaei, Pourya Mohammadi

**Affiliations:** Department of Chemistry, Payame Noor University Tehran Iran hojatveisi@yahoo.com pourya.mohammadi93@yahoo.com +98 8343724748 +98 8343724748

## Abstract

In the current research work, a new KIT-5-biguanidine-Pd(0) catalyst was prepared and applied to ultrasound-assisted Suzuki–Miyaura cross-coupling reactions using ultrasound waves at ambient temperature. The ultrasound-assisted method is a green and efficient method for C–C coupling. Many parameters of the Suzuki coupling reaction were examined, such as the irradiation time, the types of organic and inorganic bases, the types of aprotic and protic solvents, and the dosage (mol%) of catalyst. Also, the results showed that the yields from the ultrasound-assisted coupling reactions were higher than from non-irradiated reactions. The prepared catalyst was characterized *via* HR-TEM, SEM-EDX-mapping, FT-IR, ICP-AAS, BET-BJH, and XRD studies. The stability and catalytic performance of the prepared catalyst were good, and it could be reused 6 times without catalytic activity loss for the Suzuki–Miyaura cross-coupling reaction.

## Abstract 2

In the current research work, a new KIT-5-biguanidine-Pd(0) catalyst was prepared and applied to ultrasound-assisted Suzuki–Miyaura cross-coupling reactions using ultrasound waves at ambient temperature.

## Graphical abstract

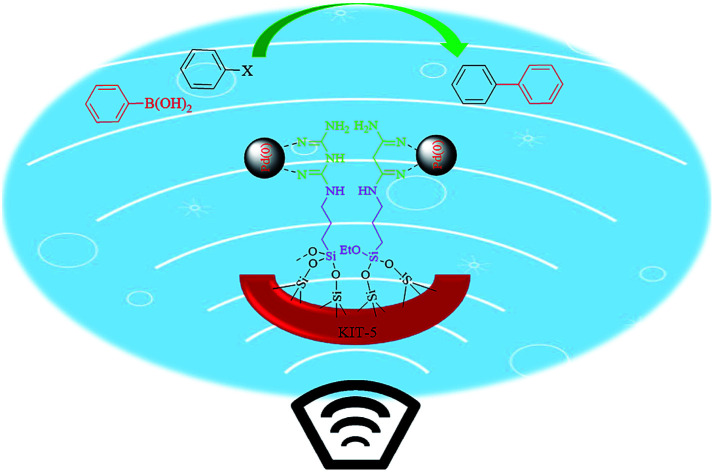

## 1. Introduction

In the last few decades, the Pd-catalyzed Suzuki–Miyaura cross-coupling reaction, as one of the reactions for C–C bond coupling, has attracted the attention of researchers. Different types of heterogeneous and homogeneous catalysts have been applied to this coupling reaction; for example, Pd complexes with phosphine ligands as homogeneous catalysts have been widely used. Nevertheless, homogeneous catalysts have basic defects, such as difficulties relating to isolation, which can cause contamination of the product; catalyst instability, leading to leaching during product purification; and considerably higher costs relating to the separation of the catalyst. On the other hand, Pd supported on a substrate, known as a heterogeneous catalyst, has many advantages, such as easy separation and good recovery from reaction mixtures, good stability, and low leaching during the reaction. Therefore, heterogeneous catalysts that involve a substrate can be separated from the reaction mixture, can be recovered from reaction mixtures and can be reused several times.^1–7^ Hence, from a green chemistry perspective, heterogeneous catalysts focusing on the utilization of Pd nanostructures are well known. Nevertheless, the high activity of Pd nanoparticles and their desire to agglomerate could lead to a decrease in their reactivity. Therefore, one useful strategy for solving these problems involves the use of many different solid supports to immobilize the Pd nanoparticles, which can modify the active site centers of the catalyst and improve the catalytic activity and reusability.^8–12^

To design an effective heterogeneous nanocatalyst, the synthesis of a support with targeted functionalization to maximize the immobilization and loading of Pd nanoparticles is necessary. One type of support used today is ordered mesoporous silica materials. These materials have unique properties, such as ordered porosity, considerably high pore volumes and high surface areas, good mechanical and chemical stability, and good surface functionalization; this means they have found use in sensing, separation, drug delivery, adsorption, and catalysis ^13,14^. Among the different types of silica ordered mesoporous materials, silica mesoporous materials that have cage-type and large-pore systems with grown porous structures in three-dimensions have been used as efficient supports for many types of heterogeneous catalysis. Also, 3D mesoporous materials have advantages *vs.* 2D materials: (1) the blocking of pores is avoided; (2) they provide highly active sites for high absorption; (3) they have the ability to absorb large molecules; and (4) they have high diffusion indices for the diffusion of reactants.^15^ Nanostructures such as SBA-16, SBA-15, and KIT-5 are examples of silica mesoporous materials that have pores with 3D cage-type structures.^16–18^ Among them, the KIT-5 mesoporous material, first introduced by Ryoo *et al.*, has 3-D close-packed cage-type *Fm*3*m* cubic symmetry, and it also has a high pore volume and surface area, and large pores.^18^ Therefore, given the reasons mentioned above, KIT-5 can be used as an efficient support for the immobilization of palladium nanoparticles. Today, it is important to prepare chemical reactants under milder conditions, such as using shorter time periods along with obtaining higher efficiencies at lower temperatures.

Ultrasound-assisted methods are efficient methods for the acceleration of many organic and inorganic reactions.^19–22^ Ultrasound, due to cavitation effects, can generate effective intensity in the reaction process, causing physical and chemical effects. Ultrasound spreads *via* a chain of expansion and compression cycles induced in a liquid medium. Accordingly, cavities are created in the liquid. These cavities grow and collapse in different stages. Eventually, they collapse furiously, producing high pressures and high local heating for very short lifetimes. The “hot spots” created have very high pressures (1000 atm) and very high temperatures (4500–5000 K); on the other hand, these spots cool down in a very short time (>1010 K s^−1^).^23–25^ These physical effects cause the dispersion of nanoparticles and can prevent them from agglomerating. Also, the separation of the materials (reactants) attached to the active surface leads to an increase in the rate and efficiency of the reaction. Given the reasons mentioned, ultrasound-assisted methods are a green methodology for the preparation of various organic and inorganic compounds under mild reaction conditions, leading to high reaction yields and lower levels of pollution.^26^

**Scheme 1:**
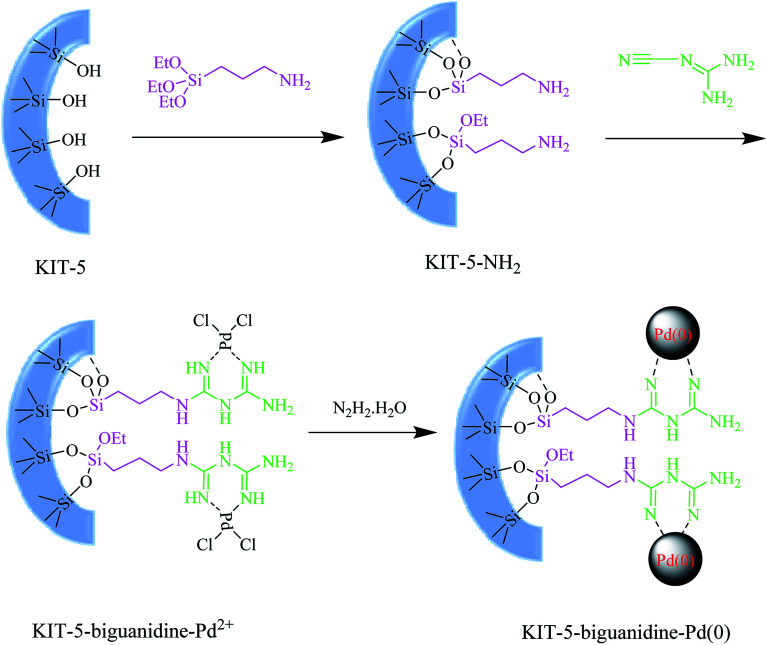
The preparation of the KIT-5-biguanidine-Pd(0) catalyst.

In continuation of our endeavors to support sustainable development using nanocatalysis,^27^ we herein introduce (Scheme 1) an ultrasound-assisted method as a simple green synthetic method using a new KIT-5-biguanidine-Pd(0) catalyst, and we applied it to the Suzuki–Miyaura cross-coupling reaction. The prepared nanocatalyst was used for the Suzuki–Miyaura cross-coupling reaction of phenylboronic acid and various aryl halides (X = Cl, Br, I) and the reactions were successfully carried out in EtOH/H_2_O as the reaction solvent at ambient temperature. This heterogeneous catalyst showed good catalytic activity and could be recovered easily *via* centrifuging; it was also capable of being re-used six times for the aforementioned transformations, with no considerable loss of activity.

## 2. Experimental

### 2.1. The preparation of KIT-5

Firstly, 8.5 g of HCl (37%), 192 g of H_2_O and 19 g of the copolymer F127 were added to a beaker, then the mixture was heated at 45 °C, 19 g of TEOS was added to it, and it was left overnight. After further aging at 100 °C for 24 h, the white precipitate was filtered and washed with distilled H_2_O and dried, and, finally, to remove the copolymer F127, it was calcined at 550 °C for 5 h.

### 2.2. The preparation of KIT-5-biguanidine

First, 1.0 g of KIT-5 and 100 mL of toluene were added to a 150 mL round-bottom flask, and the mixture was stirred at ambient temperature for 1 h. In the next step, 1 mL of 3-aminopropyl trimethoxysilane (APTMS) was added dropwise to the reaction mixture and this was then refluxed overnight under N_2_ gas. Finally, the precipitate was filtered and washed several times with ethanol (70%). Subsequently, it was dried at 60 °C overnight. Then, 0.5 g of the product from the previous step and 100 mL of acetonitrile were added to a 100 mL round-bottom flask and stirred at 40 °C for 30 min. Then, 3 mmol of cyanoguanidine was added to the reaction mixture and it was heated at 60 °C overnight. After the end of the cyanoguanidine addition reaction, the precipitate was filtered and washed several times with ethanol (70%), and it was dried at 60 °C overnight.

### 2.3. The preparation of KIT-5-biguanidine-Pd(0)

In a typical reaction, 40 mg of PdCl_2_ and 50 mL of acetonitrile was added to a 100 mL beaker and the mixture was then stirred and heated until PdCl_2_ completely dissolved (Solution A). 0.5 g of KIT-5-cyanoguanidine and 50 mL of acetonitrile was added to a 150 mL round-bottom flask and this mixture was strongly stirred at 50 °C; then, Solution A was added dropwise to this mixture. Subsequently, it was refluxed for 5 h. In the next step, 300 μL of hydrazine hydrate (N_2_H_2_·H_2_O) was added dropwise, and this mixture was then refluxed for 24 h. After the end of the reaction, the gray precipitate was filtered and washed several times with EtOH (70%) before being dried at 50 °C overnight. To determine the amount of Pd loaded on the catalyst, ICP-AAS analysis was used, and the value was estimated to be 0.3 mmol g^−1^.

### 2.4. Suzuki–Miyaura coupling reactions

The protocol for the Suzuki–Miyaura coupling reactions is described as follows: 10 mg of the KIT-5-biguanidine-Pd(0) nanocatalyst, phenylboronic acid (0.6 mmol), aryl halide (0.5 mmol) and K_2_CO_3_ (0.5 mmol) were dissolved in 5 mL of solvent under an air atmosphere. The reaction mixture was sonicated using an ultrasonic bath and the reaction progress was monitored *via* TLC. After the completion of the coupling reaction, the nanocatalyst was separated *via* centrifugation. The final coupling product was purified *via* column chromatography.

## 3. Results and discussion

### 3.1. Characterization of the catalyst

#### 3.1.1. XRD

**Fig. 1:**
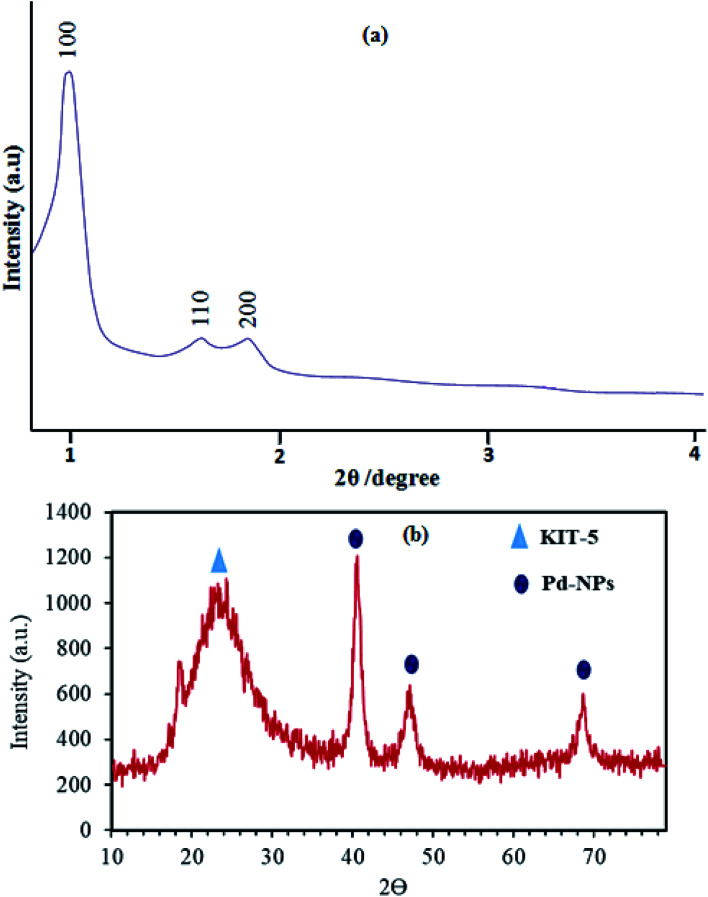
Small- (a) and wide- (b) angle XRD patterns of KIT-5-biguanidine-Pd(0).

The small-angle XRD pattern of KIT-5-biguanidine-Pd(0) is shown in Fig. 1a. Three well-resolved diffraction peaks in the 2*θ* range of 0.8–2° are observed for the prepared catalyst, which is an organic–inorganic hybrid material like its KIT-5 parent. The ordered structure of KIT-5-biguanidine-Pd(0) remained intact after functionalization, which was supported by the XRD results. The pattern features distinct Bragg peaks in the 2*θ* range of 0.8–2°, which can be indexed as the (1 0 0), (1 1 0) and (2 0 0) reflections of the two-dimensional hexagonal structure of the KIT-5 material.

Wide-angle XRD analysis was used to confirm the presence of Pd crystalline nanoparticles and their purity, and also the presence of amorphous silica from KIT-5, in the prepared KIT-5-biguanidine-Pd(0) catalyst (Fig. 1b). The XRD pattern shows diffraction peaks at 2*θ* values of 39.9°, 46.4°, and 67.5°, which relate to the planes of the Pd nanoparticles with Miller indices of (111), (200), and (220), respectively. A broad diffraction peak at about 2*θ* = 24° appeared, which is from the non-crystalline silica of KIT-5. Also, the lack of additional peaks showed that the prepared nanocatalyst was of good purity.

#### 3.1.2. FT-IR

**Fig. 2:**
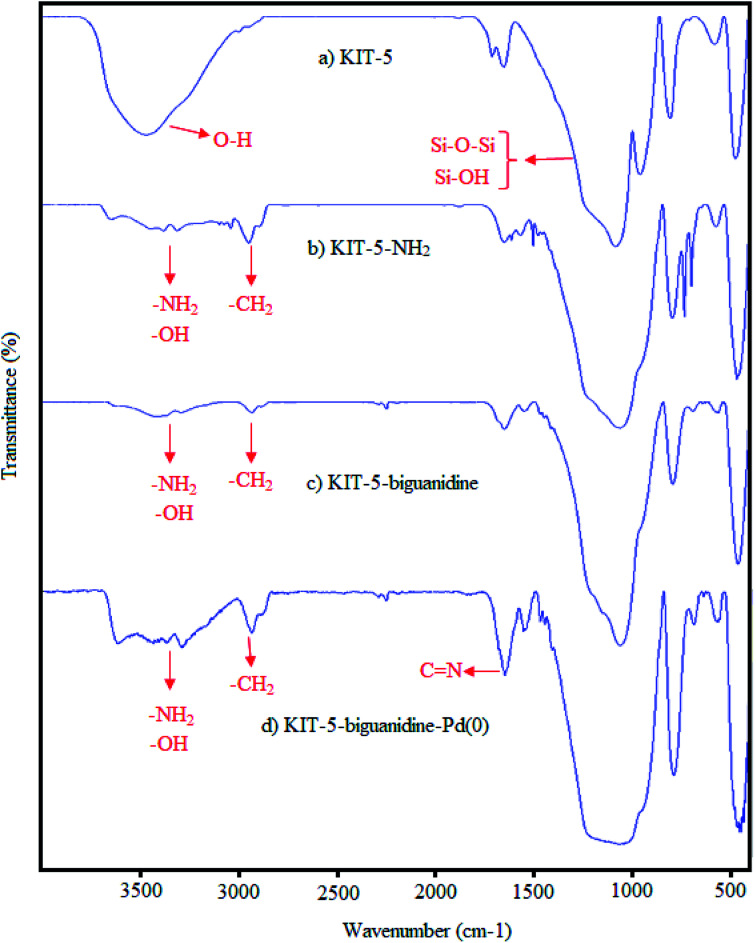
The FTIR spectra of (a) KIT-5, (b) KIT-5-NH_2_, (c) KIT-5-biguanidine, and (d) KIT-5-biguanidine-Pd(0).

FT-IR analysis was applied in order to confirm the successive synthesis steps for KIT-5-biguanidine-Pd(0) (Fig. 2). The bands at about 471 and 802 cm^−1^ were indicated to relate to the symmetric bending and stretching frequencies of Si–O–Si bonds and, also, the band at 1085 cm^−1^ can be linked to the asymmetric stretching frequency of Si–O–Si bonds (Fig. 2a). The broad peak appearing at 3436 cm^−1^ is related to the O–H stretching frequency of silanol groups (Si–OH).^28,28*a*^ Upon the functionalization of KIT-5 with APTES, new peaks appear. The weak peaks shown at 2840 and 2945 cm^−1^ correspond to C–H symmetric and asymmetric stretching frequencies, respectively. The broad peak at about 3450 cm^−1^ can result from the stretching of SiO–H and N–H bonds (Fig. 2b). In addition, the peaks that appear at 1480 and 1610 cm^−1^ can be attributed to the stretching of C–N and C=N bonds in biguanidine. The presence of these peaks indicates that the functionalization of KIT-5-NH_2_ with cyanoguanidine was done successfully (Fig. 2c). The functional group signal of C=N bonds then shifted from 1610 to 1605 cm^−1^; this change can be attributed to the coordination of biguanidine with Pd nanoparticles. This result demonstrates that the Pd nanoparticles were successfully immobilized on KIT-5 (Fig. 2d).^28*b*^

#### 3.1.3. SEM-EDX mapping

**Fig. 3:**
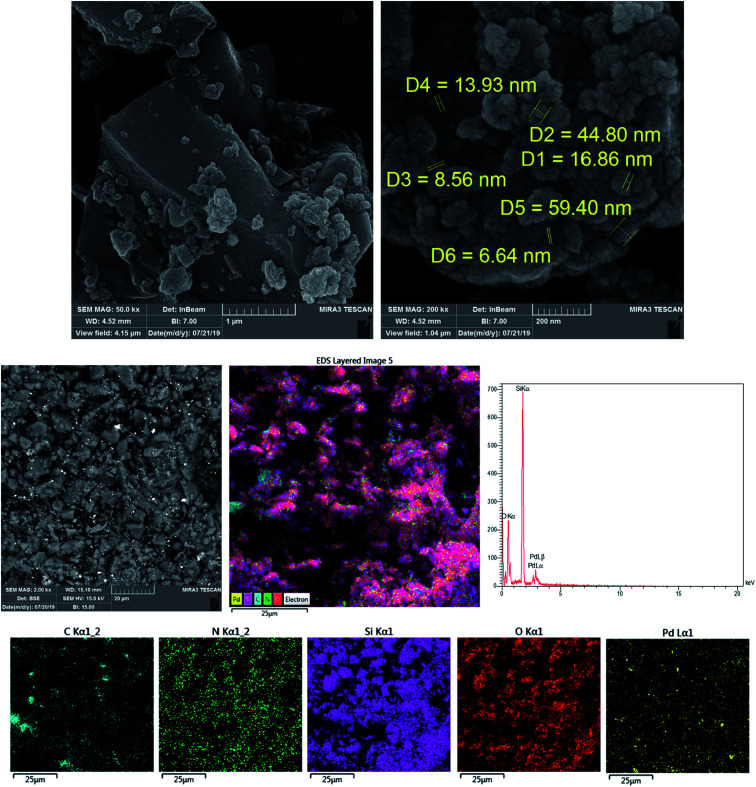
SEM imaging and SEM-EDS mapping of the KIT-5-biguanidine-Pd(0) catalyst.

The morphology of the prepared catalyst was considered *via* SEM-EDX mapping (Fig. 3). This displays that the Pd particles were aggregated and immobilized on the surface of KIT-5; as shown in the image, the Pd-NPs are spherical and their average size is about 20 nm. To elementally confirm the formation of the KIT-5-biguanidine-Pd(0) catalyst, elemental mapping was carried out *via* energy-dispersive X-ray absorption spectroscopy (EDS). Fig. 3 shows peaks from carbon, nitrogen, oxygen, silicon, and palladium, which confirms the presence of biguanidine and palladium in the KIT-5 support. From EDS analysis, the Pd content was calculated to be 0.25 mol% in the nanocatalyst.

#### 3.1.4. HRTEM-EDX studies

**Fig. 4:**
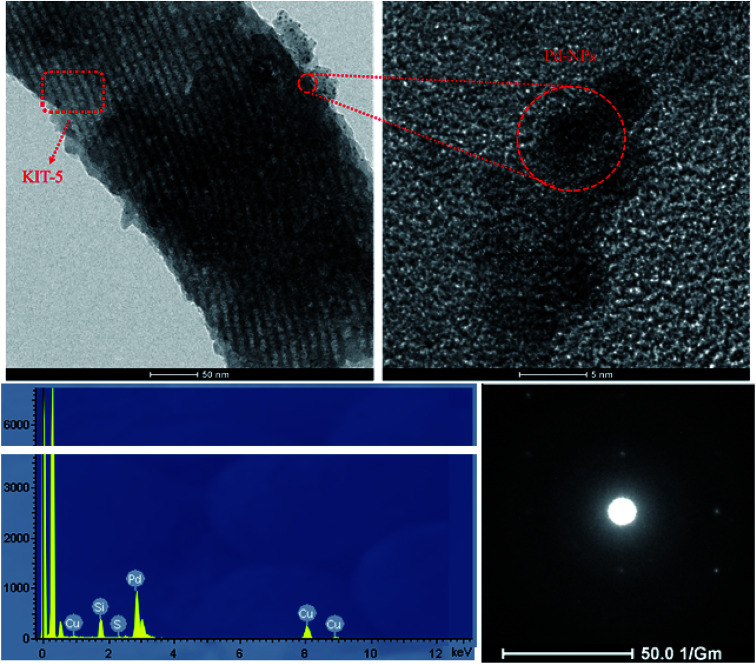
HRTEM-EDX images and the SAED pattern of the KIT-5-biguanidine-Pd(0) catalyst.

To observe and confirm the mesoporous structure of KIT-5 and the presence of Pd nanoparticles on the KIT-5-biguanidine-Pd(0) catalyst, high-resolution transmission electron microscopy-energy dispersive X-ray (HRTEM-EDX) analysis was done (Fig. 4). The mesoporous structure of KIT-5 is clearly observed with a good degree of long-range ordering. In addition, the good distribution of Pd nanoparticles on the KIT-5-biguanidine-Pd(0) catalyst can be seen in the HRTEM image. In addition, the presence of Si, N, C, and O was shown *via* this analysis. Also, the SAED pattern of the Pd nanoparticles indicated the presence of the (111), (200) and (220) lattice planes of face-centered cubic (fcc) structures, which relate to the crystalline structure of Pd-NPs.

#### 3.1.5. N_2_ adsorption–desorption

**Fig. 5:**
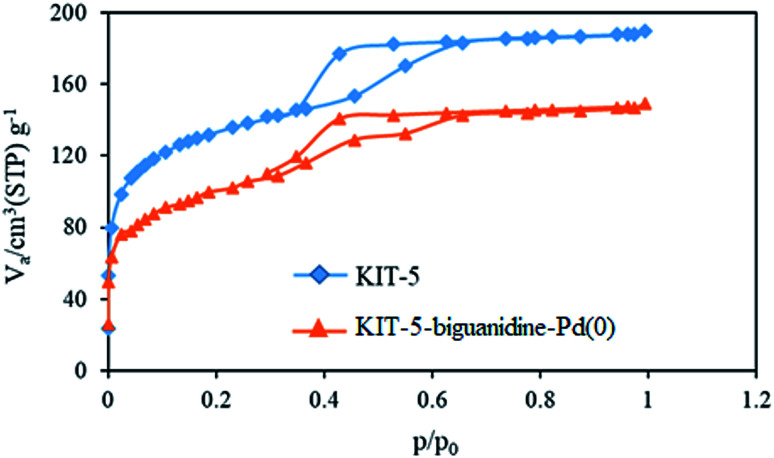
N_2_ adsorption–desorption isotherms from KIT-5 and KIT-5-biguanidine-Pd(0) samples.

N_2_ adsorption–desorption analysis of KIT-5 and KIT-5-biguanidine-Pd(0) samples is shown in Fig. 5. The textural properties obtained from this analysis are summarized in Table 1. The KIT-5 sample illustrates a type-IV adsorption isotherm and a H_2_-type hysteresis loop. The obtained results are in good agreement with the successful synthesis of mesoporous KIT-5. The pore diameter of KIT-5 is about 5.84 nm. After the functionalization of the pores and the immobilization of Pd nanoparticles, the diameter of the pores is reduced to 4.24 nm, which can be due to the filling of the pores with biguanidine and Pd nanoparticles. Also, the results show a reduction in the surface area from 578.23 to 351.31 m^2^ g^−1^, confirming that the pores are filled.

**Table 1:** A summary of the N_2_ adsorption–desorption results for KIT-5 and KIT-5-biguanidine-Pd(0) samples

Sample	*V*_p_ (cm^3^ g^−1^)	*A*_p_ (m^2^ g^−1^)	*D*_p_ (nm)
KIT-5	117.14	578.23	5.84
KIT-5-biguanidine-Pd(0)	80.71	351.31	4.24

### 3.2. Evaluation of the catalytic performance of the KIT-5-biguanidine-Pd(0) nanocatalyst towards C–C coupling reactions

**Scheme 2:**
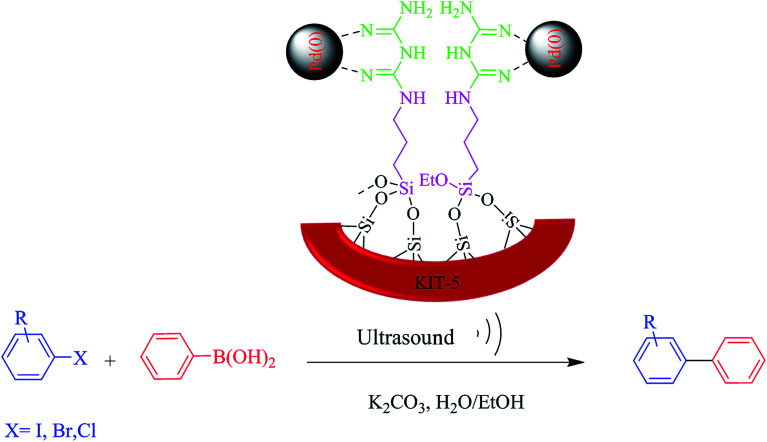
A schematic diagram of the role of the KIT-5-biguanidine-Pd(0) nanocatalyst in the Suzuki coupling reaction.

Scheme 2 schematically illustrates the catalytic role of the KIT-5-biguanidine-Pd(0) nanocatalyst for carrying out the Suzuki coupling reaction. To analyze the catalytic performance of the prepared catalyst, many parameters of the Suzuki coupling reaction were examined, such as the types of organic and inorganic bases, the types of aprotic and protic solvents, and the dosage (mol%) of catalyst. The C–C catalytic coupling was done at room temperature (25 °C) using a model reaction (the reaction of Ph-B(OH)_2_ and Ph-Br) (Table 2). At first, the effects of ultrasound on the Suzuki–Miyaura cross-coupling reaction were optimized. The obtained results show that in the presence of ultrasound, the yield of the reaction was greater than under only stirring; also, with an increase in the ultrasound time, the yield of the reaction increased and the optimized time was deemed to be 20 min (Table 2). In the process of Suzuki C–C coupling reactions, the nature of the solvent (protic and aprotic) has been proven to play an important role. For this purpose, various solvents were used, and the best solvent for this reaction was seen to be a mixture of water and ethanol (1 : 1). This mixed solvent has benefits, such as being economical and eco-friendly. Also, the most efficient base was seen to be K_2_CO_3_, and it was selected and applied. The coupling reaction was examined in the absence of nanocatalyst and no product was observed (Table 3, entry 11). It was proved that the presence of nanocatalyst was required for the coupling reaction to occur. Also, different dosages of nanocatalyst from 0.1 to 0.2 mol% were tested. Table 3 indicates that an increase in the dosage of nanocatalyst leads to an increase in the yield of the coupling reaction. Also, the results showed that the progress of the reaction with more than 0.2% mol nanocatalyst did not significantly increase the yields. So, this dosage of catalyst was selected for carrying out the Suzuki coupling reaction under favored conditions (Table 3, entry 8).

**Table 2:** Optimization of the sonication time when using the KIT-5-biguanidine-Pd(0) catalyst in the Suzuki coupling reaction

Entry	Sonication time (min)	Yield (%)
1	5	40
2	10	53
3	15	79
4	20	98
5	25	98
6	Just stirring (20 min)	38

**Table 3:** Optimization of the solvent, base and catalyst amount when using the KIT-5-biguanidine-Pd(0) catalyst for the Suzuki coupling reaction

Entry	Pd (mol%)	Solvent	Base	Time (min)	Yield (%)
1	0.25	Toluene	K_2_CO_3_	20	65
2	0.25	DMF	K_2_CO_3_	20	80
3	0.25	EtOH	K_2_CO_3_	20	82
4	0.25	H_2_O	K_2_CO_3_	20	55
5	0.25	EtOH/H_2_O	Et_3_N	20	93
6	0.25	EtOH/H_2_O	NaOAc	20	80
7	0.25	EtOH/H_2_O	K_2_CO_3_	20	98
8	0.20	EtOH/H_2_O	K_2_CO_3_	25	85
9	0.15	EtOH/H_2_O	K_2_CO_3_	30	74
10	0.10	EtOH/H_2_O	K_2_CO_3_	30	62
11	0.0	EtOH/H_2_O	K_2_CO_3_	30	0
12	0.25	EtOH/H_2_O	No base	20	Trace

### 3.3. Investigation of functional group effects on C–C coupling

**Fig. 6:**
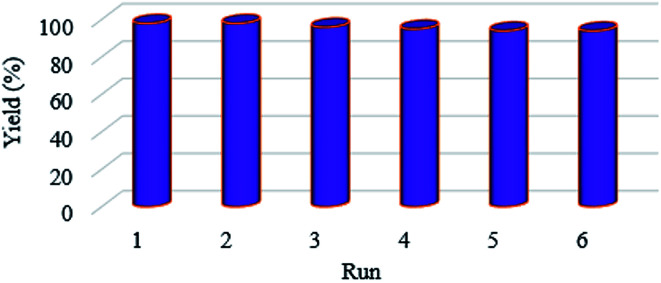
Reusability of the KIT-5-biguanidine-Pd(0) catalyst.

The Suzuki C–C coupling reactions of variously substituted aryl halides with electron-withdrawing and electron-donating functional groups with phenylboronic acids was done in the presence of 0.2 mol% of the nanocatalyst under optimized conditions (Table 4). Also, under the optimized conditions, the coupling reactions of phenylboronic acid with different types of aryl halides, such as phenyl chlorides, phenyl bromides, and phenyl iodides, were carried out (Table 4, entries 1–17). The results illustrated that aryl halides with an electron-withdrawing functional group showed more activity than those with an electron-donating functional group in the *para*-position. For a more accurate evaluation of the prepared catalyst, a wide range of activated and deactivated aryl halides was examined. According to the results obtained, medium yields were observed under the optimized reaction conditions due to the C–Cl bonds in aryl chlorides being stronger than the C–Br bonds in aryl bromides, so the yields from the coupling of aryl bromides were greater than those from aryl chlorides (Table 4, entries 2–3, 5–6 and 8–9). As expected, on the basis of the results obtained, aryl boronic acids with electron-donating functional groups showed excellent yields (Table 4, entries 16–17). To test the stability of the prepared catalyst, recovery tests were carried out that showed that it could be used efficiently for 6 runs without significant loss of activity (Fig. 6).

**Table 4:** Investigation of the effects of functional groups when using the KIT-5-biguanidine-Pd(0) catalyst for the Suzuki coupling reaction

Entry	RC_6_H_4_X	R_2_C_6_H_4_B(OH)_2_	X	Time (min)	Yield (%)
1	H	H	I	15	98
2	H	H	Br	20	98
3	H	H	Cl	60	72
4	4-CH_3_	H	I	15	96
5	4-CH_3_	H	Br	30	90
6	4-CH_3_	H	Cl	60	68
7	4-COCH_3_	H	I	20	92
8	4-COCH_3_	H	Br	45	93
9	4-NO_2_	H	Cl	60	80
10	4-OCH_3_	H	I	20	94
11	4-OCH_3_	H	Br	45	92
12	4-NO_2_	H	I	20	95
13	4-NO_2_	H	Br	60	92
14	4-CH_3_	H	I	20	88
15	4-NO_2_	4-OCH_3_	I	60	92
16	H	3-OCH_3_	Br	60	90
17	H	2-OCH_3_	Br	60	87

The activity and efficiency of the KIT-5-biguanidine-Pd(0) catalyst were compared to various catalysts for the Suzuki coupling reaction (Table 5). As shown, the prepared catalyst demonstrated good yields and moderate conditions compared to other reported catalysts.

**Table 5:** Comparison of the KIT-5-biguanidine-Pd(0) catalyst with various catalysts for the Suzuki coupling reaction

Entry	Catalyst (mol%)	Conditions	X	Time (h)	*T* (°C)	Yield (%)	Ref.
1	PdCl_2_(MeCN)_2_ (0.02 mmol)	K_2_CO_3_, H_2_O/DMF	Br	5 min	r.t.	95	29
2	SiO_2_-pA-Cyan-Cys-Pd (0.5)	K_2_CO_3_, H_2_O	I, Br	5, 5.5	100	95, 88	30
3	Pd–BOX (2)	K_2_CO_3_, DMF	I	6	70	100	31
4	Bis(oxamato)palladate(II) complex (5)	Et_3_N, *n*-Bu_4_NBr	I, Br	2	120	78, 65	32
5	Pd-isatin Schiff base-γ-Fe_2_O_3_ (0.5, 1.5)	Et_3_N, solvent-free	I, Br	0.5, 0.75	100	95, 90	33
6	NHC–Pd(ii) complex (0.2)	K_3_PO_4_·3H_2_O, H_2_O, TBAB	I, Br	5, 6	40	98, 90	34
7	γ-Fe_2_O_3_–acetamidine–Pd (0.12)	Et_3_N, DMF	I, Br	0.5, 0.5	100	96, 96	35
8	Pd_3_(dba) (1)	K_3_PO_4_, THF	Br	24	80	77.7	36
9	KIT-5-biguanidine-Pd(0)	K_2_CO_3_, H_2_O/EtOH	I, Br	15 min	r.t.		This work

## 4. Conclusions

In this work, an ultrasound-assisted simple green synthetic method was introduced, using a new KIT-5-biguanidine-Pd(0) catalyst, and it was applied to the Suzuki–Miyaura cross-coupling reaction. To analyze the catalytic performance of the prepared catalyst, many parameters of the Suzuki coupling reaction were examined, such as the types of organic and inorganic bases, the types of aprotic and protic solvents, and the dosage (mol%) of catalyst. On the basis of the results observed, a temperature of 25 °C, the use of ethanol/water (1 : 1) as the solvent, and a nanocatalyst amount of 0.2 mol% were selected for carrying out the Suzuki coupling reaction under favorable conditions. This heterogeneous catalyst system showed excellent catalytic performance and can be recovered easily *via* centrifuging; it can also be reused 6 times for the aforementioned transformation with no considerable loss of activity.

## Conflicts of interest

The authors report no conflicts of interest related to this work.

## Supplementary Material
